# Inhibition of triple negative breast cancer-associated inflammation and progression by N- acylethanolamine acid amide hydrolase (NAAA)

**DOI:** 10.1038/s41598-022-26564-6

**Published:** 2022-12-23

**Authors:** Othman Benchama, Michael S. Malamas, Kulkarni Praveen, Elizabeth C. Ethier, Mark K. Williams, Alexandros Makriyannis, Hava Karsenty Avraham

**Affiliations:** 1 Center of Drug Discovery, Northeastern University, Boston, MA 02115 USA; 2 Center for Translational Neuroimaging, Northeastern University, Boston, MA 02115 USA; 3 Mineral Logic LLC, Kalamazoo, MI 49048 USA

**Keywords:** Breast cancer, Cancer metabolism, Drug discovery

## Abstract

Triple-negative breast cancer (TNBC) is associated with high mortality due to the high expression of pro-inflammatory cytokines and lack of targeted therapies. N-acylethanolamine acid amidase (NAAA) is an N-terminal cysteine hydrolase that promotes inflammatory responses through the deactivation of Palmitoylethanolamide (PEA), an endogenous bioactive lipid mediator. Here, we examined NAAA expression in TNBC cells (MDA-MB-231 and MDA-MB-BrM2 cells) and the effects of NAAA inhibition on TNBC tumor growth, using a selective NAAA inhibitor AM11095 (IC_50_ = 20 nM). TNBC cells expressed elevated levels of full-length and splice mRNAs naaa variants. TNBC cells also express the N-acyl ethanol amides and elevated levels of the two fatty acid cores arachidonic (AA) and docosahexaenoic (DHA). PEA or AM11095 inhibited the secretion of IL-6 and IL-8, reduced the activation of the NF-kB pathway, decreased the expression of VEGF and Placental growth factor (PLGF) in TNBCs, and inhibited tumor cell migration in vitro. Using cellular magnetic resonance imaging (MRI), body images of mice administered with human MDA-MB-BrM2 cells treated with AM11095 showed a significant decrease in tumor numbers with a lower volume of tumors and increased mice survival. Mice untreated or treated with vehicle control showed a high number of tumors with high volumes in multiple organs. Thus, NAAA inhibition may constitute a potential therapeutic approach in the management of TNBC-associated inflammation and tumor growth.

## Introduction

Triple negative breast cancer (TNBC) is a heterogeneous disease associated with poor patient survival due to high metastasis rate and lack of targeted therapies^1–3^. Patients with TNBC have a brief period of remission, resulting in rapid recurrence of TNBC and/or TNBC metastatic disease. TNBC represents 15% of breast carcinomas and is defined by the absence of the three main breast cancer biomarkers: lack of expression of estrogen receptors and progesterone receptors and lack of amplification or overexpression of HER-2 (also known as ERB2). Important parameters of TNBC biology include high proliferative activity, increased immune cell infiltrate, a basal- like and a mesenchymal phenotype, a deficiency in homologous recombination partly linked to a loss of BRCA1 function and the Androgen receptors. Due to the aggressive biology of TNBC, there is an urgent need to develop novel therapeutic strategies against TNBC growth and metastasis.

Emerging studies strongly suggest that abnormal metabolism is linked to chronic inflammation which is most likely to modify tumor microenvironment leading to promotion of tumorigenesis cascades^4–6^. However, it is unknown whether chronic inflammation pays a role in TNBC initiation. Cancer inflammation is associated with elevated inflammatory mediators, indicator of poor prognosis in patients with cancer^6–13^. The connection between inflammation and cancer is based by the observations of uncontrolled cell proliferation at the site of inflammation and the existence of inflammatory cells at the tumor site. The inflammatory mediators and the reactive nitrogen oxygen species induce the activation of NF-kB and cyclooxygenase-2 (COX-2) pathways, facilitating inflammation-mediated tumorigenesis. Persistent inflammation may induce altered expression of oncogenes and tumor suppressor genes. When acute inflammatory response is developed to chronic inflammation, this process may lead to the initiation of tumors.

Cancer cells synthesize 95% of fatty acids (FAs) de novo^14–18^. Tumor cells employ de novo lipogenesis and use exogenous FAs, including exogenous palmitate, which are incorporated into structural and oncogenic glycerophospholipids, sphingolipids and ether lipids in tumor cells^19–21^. The survival of tumor cells is facilitated by fatty acid synthesis (FAS) over-expression, which provides survival advantages to tumor cells and tumor resistant cells. Thus, targeting lipid metabolism should improve treatment responses in cancer cells.

Fatty acid palmitoylethanolamide (PEA) is a bioactive lipid that potently inhibits pain and inflammation by activating nuclear receptors, the peroxisome proliferator-activated receptors (PPAR-α)^22–25^. PEA is an endogenous agonist for PPAR-α^30,31,42^. PPARs play important roles in regulating lipids, and can promote eNOS activation, regulate immunity and inflammation responses^26^. PEA exerts it effects through NF-kB, leading to inhibition of inflammation^27,28^. PEA is preferentially degraded by N-acylethanolamine hydrolyzing acid amidase (NAAA)^29,30^. Recent reports suggest that PEA and related signaling bioactive lipids are increasingly recognized as playing a key role in down regulating cancer growth^19,23,26^. Because of its multi-faceted activity, PEA, and its hydrolytic enzyme NAAA have been considered important lipid modulators since they are able to exert anti-inflammatory effects. However, to date, no proof about their anti-tumorigenic effects on TNBCs has been provided yet.

A quantitative study on splice variants of N-acylethanolamine acid amidase in human prostate cancer cells was reported^35^. These truncated forms were detected as catalytically inactive precursor proteins, but not as mature forms^35^. A wide distribution of multiple variants of NAAA mRNA in various human cells suggest that the proteins from some variants are catalytically inactive.

Accumulated studies demonstrated the benign side-effect profile of PEA, including six clinical trials in a total of 4,000 people^32,33^. These were performed by PEA administration as a therapy for influenza and the common cold virus infection (Impulsin) and as a dietary supplement for pain (Normast). However, PEA cannot serve as a treatment option due to its poor pharmacokinetics properties. Thus, the current approach is to enhance the endogenous levels of PEA, through inhibition of its catalytic enzyme NAAA, using specific NAAA inhibitors.

In this study, we hypothesized that enhancement of endogenous levels of PEA, via inhibition of its catalytic enzyme NAAA, should lead to inhibition of pro-tumor signaling cascades in TNBC, resulting in reduced inflammation and tumor growth. We employed a selective NAAA inhibitor AM11095 with high specificity and potency toward NAAA with an IC_50_ = 20 nM and with no overt signs of toxicity. Several human cell lines were employed: Non-tumorigenic MCF-10A, triple positive breast cancer MCF-7 cells, TNBC human cell line (MDA-MB-231) and human brain-seeking TNBC cell line (MDA-MB-BrM2). We found that AM11095 inhibited the secretion of inflammatory cytokines such as IL-6 and IL-8, and the angiogenic factors VEGF-A and PLGF. In vitro studies showed that AM11095 inhibited TNBC cell migration. Using MRI, body images of mice treated with AM11095 revealed that the average numbers and volume of tumors were significantly reduced, and mice survival increased, while mice treated with vehicle control exhibited high number of tumors in multiple organs. Taken together, this study reveals insights into the role of NAAA in TNBC tumor associated inflammation and tumor progression.

## Materials and methods

### Ethics statement

The animal study protocol was approved by The Institutional Animal Care and Use Committee of Northeastern University Institutional Animal Care and Use Committee (IACUC), protocol number 20-0624R, according to the Public Health Service (PHS) Policy on Humane Care and Use of Laboratory Animals and the Guide for the Care and Use of Laboratory Animals (NIH), for the protection of Vertebrates Used for Scientific Purposes (Scientific Procedures) Act 1986 as well as the ARRIVE guidelines for reporting in vivo experiments in animal research. All animal studies were performed in Northeastern University (Boston).

### Breast cancer cell lines

Normal immortalized breast epithelial cells (MCF-10A), and the breast cancer cell line MDA-MB-231 were purchased from American Type Culture Collection (ATCC, Manassas, VA) and maintained according to ATCC’s protocol. The breast tumor metastatic brain variant MDA-231-BrM2 tagged to GFP which specifically form metastatic brain tumors^34^. All breast cancer cell lines were maintained in RPMI-1640 medium supplemented with 10% FBS (Atlanta Biologicals, Norcross, GA), 2.9 mg/ml Glutamine, and 100 U/ml Penicillin/Streptomycin, and incubated in a 5% CO_2_ incubator at 37 °C.

#### qPCR: mRNA expression for NAAA GENE VARIAnts

Human NAAA mRNA expression in MCF10A, MCF-7, MDA-MB-23, and MDA-MB-BrM2 cells were analyzed as described^35^. NAAA mRNA was previously reported to contain multiple 3’-end spliced variants that consist of four major splice variants comprised of exons 1–11, exons 1–10 and 12, exons 1–9 and 12, and exons 1–8 and 12, respectively (a1, a2, b2, and c2), as described^35^. Briefly, the quantitative polymerase chain reaction methods to individually quantify these NAAA variants as well as measure all the variants simultaneously, as detailed by Dr. Sakura’s group^35^. The Naaa PCR primers sequences used in this study are shown in Supplemental Table S1^35^.

#### NAAA selective inhibitor AM11095

The Center for Drug Discovery (CDD) at Northeastern University (NEU) has generated a series of novel isothiocyanate derivatives that inhibit NAAA in a potent and selective manner including the NAAA inhibitor AM11095^30^. AM11095 is potent NAAA inhibitor with an IC_50_ = 20 nM, does not bind to human CB2 or to the CB1 receptors, and is selective against NAAA as it does not inhibit hydrolytic enzymes FAAH, MGL or the ABHD6. The results of the fluorescent inhibition assay demonstrated an IC_50_ > 1 µM (3pts) for all the aforementioned enzymes.

#### Fluorescent NAAA Inhibition Assay

The NAAA assay was performed using purified human NAAA and the fluorogenic substrate *N*-(4-methyl coumarin) palmitamide (PAMCA), which is hydrolyzed by NAAA to palmitic acid and the fluorescent 7-amino-4-methyl coumarin (AMC). The compounds were first screened in a three-point assay at concentrations of 100, 10 and 1 μM. Those compounds that produced greater than 50% inhibition at a concentration of 1 μM were subjected to the same NAAA assay using eight different concentrations of the compound to generate full inhibition curves. The compounds were incubated at 37 °C with shaking on a platform, with the enzyme in the assay buffer for 15 min before addition of the substrate and initiation of fluorescent readings. The IC_50_ values reported are the average of three experiments ± S.D.

#### Cell viability Studies

PEA or AM11095 were incubated with cells at 100 nM, 250 nM, and 500 nM concentrations for 72 h, followed by an MTT assay to determine the cell viability. The MTT assay was performed according to the manufacturer’s protocols supplied in the cell proliferation assay kit from Abcam Cat# ab211091.

### Measurements of PEA levels

Treated and untreated cells were detached and harvested, washed three times with 1X PBS and lysed using radioimmunoprecipitation assay (RIPA) buffer containing 1X protease and phosphatase inhibitors. Samples were normalized by diluting the cell lysate to the same total protein concentration; Internal standards were added, and protein was precipitated using 50 ul PBS and 500 ul Acetone (both ice cold), vortexed, centrifuged for 5 min at 12,000 rpm, transferred supernatant to new vials and discard pellet, then evaporated under nitrogen flow at 30 °C (until 250 ul are left in vials). Followed by liquid/liquid extraction using 100 ul PBS (ice cold), 250 ul MeOH and 500 ul CHCl_3,_ vortexed, centrifuged for 5 min at 12,000 rpm, then transferred entire bottom layer to new vials, evaporated under nitrogen flow at 30 °C until completely dry, reconstituted with 200 ul of EtOH, centrifuged for 5 min at 12,000 rpm at 4 °C, and transferred supernatant to HPLC vials.

#### LC–MS analysis for endocannabinoids

Chromatographic separation was achieved using a Higgins Analytical Haisil C18 column (0.5 × 50 mm, 5 mm) on an ABI 4000 Q-Trap mass spectrometer with a Tempo nano-LC on the front end (Applied Biosystems Incorporated; Framingham, MA)^48^. The mobile phase consisted of 95/5 water/acetonitrile and 95/5 acetonitrile/water, with 0.1% formic acid in both, in the following gradient: initial conditions are held at 30% A for 30 s, increased linearly to 100% B and held from 0.75 to 4 min, then returning to initial conditions by 4.5 min (flow rate = 10 µl/min); the autosampler was kept at 4 °C to prevent analyte degradation. Eluted peaks were ionized via electrospray ionization (ESI) in SRM mode. Deuterated internal standards were used for standard curves of cannabinoid species, and their levels per 1.5 × 10^7^ cells were determined.

##### PEA detection using LC–MS instrument

Chromatographic separation was achieved using Agilent Zorbax SB-CN column (2.1 × 50 mm, 5 μm) as described by Williams et al., (see reference 50). Hardware consisted of a Finnigan TSQ Quantum Ultra triple quad mass spectrometer with either an APCI or ESI source and an Agilent 1100 front end. The mass spectrometer was run in the APCI positive mode for detection of the ethanolamides and glycerol. The mobile phase consists of 10 mM ammonium acetate, pH 7.3 (A) and methanol (B) in the following gradient: initial conditions were held at 10% B for 1 min then increased linearly from 60 to 75% B from 1.5 to 10 min, then to 95% B within half a minute and holding for 2.5 min before returning to initial conditions. The run time is 15 min at a flow rate of 0.5 mL/min. The reagent gas used is N_2_, while the vaporizer and capillary temperatures were 350 °C and 250 °C, respectively; the coronal discharge current was set at 6μa. The collision pressure is 1mtorr, the sheath gas and auxiliary gas were set at 25 and 5 respectively, and the source CID was set at 8. The mass spec is in SRM mode and deuterated internal standards were used for the quantitation of the PEA endogenous ligands.

##### Milliplex Analysis

Cells at the concentration of 3.5 × 10^5^ cells/ml were plated in 0.5% FBS DMEM media and treated with either vehicle control or with AM11095 (250 nM) for 72 h. Then, the supernatants were collected, centrifuged to remove any debris, samples were normalized based on the protein concentrations, and analyzed by the Milliplex immunobeads panel technology (EMD Millipore’s MILLIPLEX MAP Human Cytokine/Chemokine kit) for the simultaneous quantification of the human cytokines and chemokines including IL-6, IL-8, TNF-Beta, and IFN-Gamma.

##### Analysis of NF-κB pathway activation

We examined the NF-κB signaling pathway activation using the Milliplex map NF-κB signaling magnetic beads kit 6-plex cell signaling assay (Millipore Sigma, 48-630MAG). This assay enables the simultaneous relative quantitation of multiple phosphorylation and total pathway proteins which include the following analytes: c-Myc, FADD (Ser194), IKBalpha (Ser32), IKKalpha/beta (Ser177/Ser181), NF-κB (Ser536) and TNFR1. Untreated or treated cells were lysed in Milliplex map lysis buffer containing protease inhibitors. Each lysate was diluted in assay buffer according to manufacturer’s instructions and frozen at − 80 °C until the protein measurements were performed. Samples were processed according to the assay protocol, and the median fluorescence intensity (MFI) was measured with the Luminex system.

##### Cell invasion

Treated and untreated cells were added to the top chamber and allowed to migrate through the filter membrane coated with Basement Membrane Extract (BME), which mimics the extracellular matrix, to the bottom chamber for 72 h. This assay was done according to the 96 well invasion assay from Trevigen Catalog# 3455-096-K.

##### Cell Migration

Treated and untreated cells were added to the top migration chamber and allowed to migrate through the permeable membrane to the bottom for 72 h. This assay was done according to the 96 well migration assay from Trevigen cat# 3465-096-k protocol.

##### Knockdown of NAAA by small interfering RNA (siRNA)

Contraposing the different encoding regions of NAAA transcript, small interfering RNA (siRNA) were designed and chemically synthesized (Dharmacon Inc.). On- Target plus Human NAAA siRNA-SMARTpool (see Supplemental Table 2 for more information on the siRNA NAAA sequences) were employed to silence Naaa gene. Cells were placed in six-well plates and then transfected with NAAA siRNA-SMARTpool using Lipofectamine 2000 reagent (Invitrogen) following the manufacturer’s instructions. Each experiment was repeated three times.

##### Cytokines and chemokines analysis using human antibody array

The Human Cytokine Array kit (R&D Systems, Catalog # ARY005B) detecting 36 human cytokines, chemokines, and acute phase proteins simultaneously was employed to detect specific targets that are affected by AM11095. This is a membrane-based sandwich immunoassay. The captured proteins were visualized using chemiluminescent or Laser Scanner. The targets include IL-6 and IL-8.

##### Human tumor cell growth in mice

Female (6wks) Nude mice were purchased from Jackson Laboratories (Bar Harbor, ME). The mice were housed at an AAALAC-accredited facility at Northeastern University, Boston. The mice were managed in accordance with the animal care policy of Northeastern University. Mice were euthanized by CO_2_ inhalation in the end of the experiments following treatment and tumor samples were harvested for further study as described below. For tumor cell administration, we employed Corning Matrigel Matrix as a scaffold for supporting the implantation of tumor cells in vivo. This matrix is a solubilized basement membrane preparation extracted from Engelbrecht-Holm-Swarm (EHS) mouse tumor rich in ECM proteins. Its major component is laminin, followed by collagen IV, heparan sulfate proteo-glycan, and entactin (Cat. Nos. 354234). Briefly, GFP-MDA-MB-BrM2 tumor cells (5 × 10^4^ cells) were mixed with Corning Matrigel Matrix (according to the protocol provided by the company) and were administered into female Nude mice intraperitoneally (IP) (using needle size 25 g 5/8″ needle). Mice (10 mice per group per treatment, experiment was repeated twice) were either untreated, or treated with vehicle control or with AM11095 at 30 mg/Kg, twice a week for 4 weeks. AM11095 was prepared in a vehicle solution consisting of a 1:1:18 mixture of ethanol, Tween 80 (Sigma) and saline. Mice were monitored daily. At the endpoint of the experiment at Day 28, all mice were analyzed by MRI as described below.

##### Magnetic resonance imaging

MRI was performed at our Preclinical MRI core facility at Northeastern University on a Bruker BioSpec 7 T/20-cm USR MRI spectrometer controlled by ParaVision 6.0.1 software, as described^36^. Radio frequency signals were sent and received with a custom quadrature volume coil built into the animal restrainer (Animal Imaging Research, Holden MA.). Mice, at Day 28 (ENDPOINT of the experiment), were placed into the coil. The T1-weighted coronal images of the abdominal region were collected using a fast low angle shot (FLASH) sequence. The imaging parameters included a time to repeat (TR)/time to echo (TE) of 425 ms/5 ms and flip angle (FA) of 30°. With a data matrix of 400 × 220 × 27 and a field of view of 40 × 20 × 19 mm, the size of each voxel was 0.1 × 0.1 × 0.7 mm. Physiological parameters including respiratory rate and oxygen saturation were monitored during imaging sessions. The T2-weighted images were collected in the same orientation and field of view. Care was taken when setting up the scans to ensure that T2 images geometry overlapped the T1 weighted images. T2 weighted images were collected using RARE (Rapid Acquisition with Relaxation Enhancement) sequence with TR/TE of 3000/48 ms. The geometrical parameters were the same as T1 weighted images.

##### Imaging analysis

Image preprocessing and analysis were performed with a combination of ITK-SNAP 3.6.0 (http://www.itksnap.org/pmwiki/pmwiki.php) and MATLAB software. T1 and T2 weighted image were extracted into nifti format and rescaled to the original intensity measurements (divided by the receiver gain and multiplied by the scaling factor) for each subject. The tumors were segmented in ITK-scan with T1 weighted MRI images using snake’s algorithm. ITK-SNAP’s default setting was used Finally, segmentation was inspected and if needed then manually corrected for any errors and overfilling. The volume of tumor was computed from segmentation images for further analysis. Statistical analyses were performed using Prism 6 (GraphPad). Raw data points are presented as mean ± SD and two-way ANOVAs were used for comparison. The parameters extracted from the fitting curves were compared using a two-sample t test. *P* < 0.05 was considered statistically significant.

##### Statistical significance of MRI imaging

Statistical analyses were performed using Prism 6 (GraphPad). Raw data points are presented as mean ± SD and two-way ANOVAs were used for comparison. The Bonferroni correction post-hoc analysis was then used to calculate the *P* values.

##### Immunostaining and hematoxylin–eosin (H&E) staining

For tumor sections analysis, immunostaining and hematoxylin–eosin staining were performed by iHisto (www.ihisto.io; Salem MA). All evaluations were performed with no knowledge of the study protocol (cell line, time point, tumor location, etc.). For tumor burden, paraffin-embedded tumor tissues were sliced in 4 μm sections. The tissues were stained with hematoxylin and eosin for measurement of tumor burden. Images of the sections were taken by a digital camera under a dissecting microscope and serial non-overlapping fields were captured to Scion Image Beta 4.03 Win software was used to measure the areas of the sections occupied by tumors.

### Multiplex immunofluorescence staining

Immunofluorescence staining was performed by iHisto Inc. (ihisto.io) Samples were processed, embedded in paraffin, and sectioned at 4 μm. Paraffin sections were then deparaffinized and hydrated using the following steps: 15 min in xylene twice; 5, 5, and 5 min in 100%, 100%, and 75% ethanol, respectively; and 5 min in PBS at room temperature repeated three times. Heat-induced epitope retrieval (HIER) was achieved by pressure cooker (SB Bio) for 15 min with sodium citrate buffer and 20 min of cooling down at room temperature. Sections were then washed with PBS three times, and blocked with 3% bovine serum albumin for 30 min. The sections were incubated with the primary antibody Anti-IL6 (GB11117, 1:100) and anti-VEGF (R&D systems AF293, 1–100) overnight at 4 °C. Sections were rinsed with PBS and incubated with Donkey anti-Goat AF488 (Invitrogen, A32814, 1:500) for 1 h at room temperature followed by 3 times of wash. Subsequently, the sections were with incubate with Goat anti-rabbit AF647 (Invitrogen, A21245, 1:500) and Goat anti-mouse AF555(Invitrogen, A21424, 1:500) for another 1 h. Lastly, quench autofluorescence with Sudan Black and stain DAPI. Whole slide scanning (40×) was performed on a Panoramic midi scanner (3D histech).

### Statistical analysis

All values are expressed as the mean ± SEM of the mean and are representative of two to three independent experiments as indicated. *P* values were calculated using unpaired two-tailed Student’s tests, built in the GraphPad Prism Software for evaluation of statistical significance.

## Results

### NAAA expression in TNBC cells

**Figure 1:**
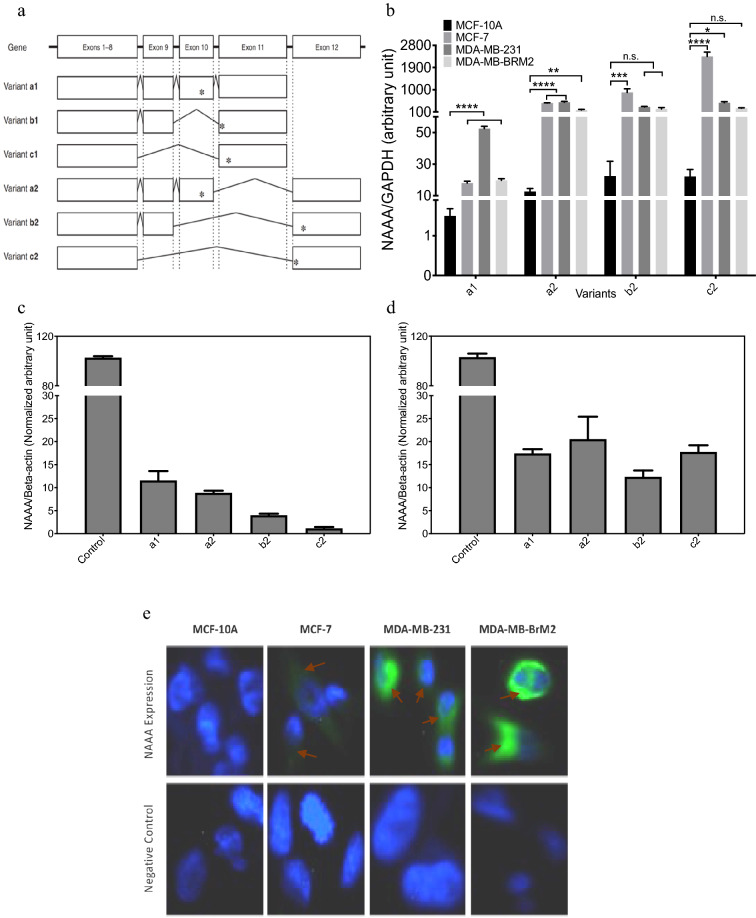
NAAA slice variants gene and protein Expression using qPCR and Immunofluorescence. (**a**) Schematic representation of NAAA splice variants and the sequences of PCR primers for NAAA were used as described in methods and elsewhere^35^. (**b**) qPCR results of the main variants of NAAA mRNA expression in MCF10A, MCF7, MDA-MB-231 and MDA-MB-BrM2 cells. The results are normalized to the levels of GAPDH mRNA expression. Values are expressed as means ± S. D (n = 3). (**c**) Expression of NAAA gene variants in siRNA treated MDA-MB-231 cells. The results are normalized to the levels of GAPDH mRNA expression. Values are expressed as means ± S. D (n = 3). (**d**) Expression of NAAA gene variants in siRNA treated MCF-7 cells. The results are normalized to the levels of GAPDH mRNA expression. Values are expressed as means ± S. D (n = 3). (**e**) Immuno-Fluorescence staining showing NAAA protein expression (Green color) and DAPI nucleus staining in blue. Isotype antibody was used for negative controls.

First, we examined the human NAAA mRNA which was previously reported to consist of multiple 3’-end splice variants^35^. We performed qPCR to quantify these NAAA variants as well as collectively measure all the variants (Fig. 1a, and Table S1). The total levels of NAAA mRNAs in breast cancer cells were significantly higher as compared to normal breast epithelial cells MCF-10A cells (Fig. 1b). Based on the qPCR results (Fig. 1b), a significant increase in the expression of full length and splice variants of NAAA in human breast cancer cells as compared to normal breast epithelial cells were observed. Specifically, breast cancer cells express significant levels of A2, B2, C2 while normal MCF-10A cells has extremely low expression of these isoforms (Fig. 1b). One way ANOVA analysis was performed for each isoform followed by Dunnett method post-hoc analysis to compare the expression to MCF-10A cells. Further, when cells were treated with siRNA-naaa gene silencing, a reduction in the expression of NAAA variants (A1, A2, B2 and C2) were detected in MDA-MB-231 and MCF-7 cells (Fig. 1c + d).

Next, the expression of NAAA protein in TNBC cells was performed by immunofluorescence staining. NAAA protein expression was abundant in MDA-MB-231 and MDA-MB-BrM2 cells, less in MCF-7, and not detected in MCF-10A cells (Fig. 1e). Based on these results, we decided to focus on MDA-MB-231 and MDA-MB-BrM2 cells.

### Quantitative analysis of the N-acyl ethanolamides and acids in breast cancer cells by LC-atmospheric pressure chemical ionization-MS

Standard curves for each of the compounds were linear with a regression value of > 0.99. Extraction efficiencies were in the 90–100% range for each compound and deuterated standard. Calibration curves were prepared ranging from 1 pg/ul to 1000 pg/ul for the Q-Trap analysis as compared to 5–500 pg/ul for the standard method performed on the Quantum. We examined the expression of the N-acyl ethanolamides, Palmitoyl ethanolamide (PEA) and Oleoyl ethanolamide (OEA) in MCF-10A cells and breast cancer cells by LC-Atmospheric Pressure Chemical Ionization-MS (LC-APCI-MS-MS)^48^. PEA and OEA were detected in MCF-10A cells (0.336 and 0.061 ng/10^6^ cells, respectively) and their expression levels were lower in tumor cells (Table 1). However, the two fatty acid cores arachidonic (AA) and docosahexaenoic (DHA) were elevated in tumor cells as compared to MCF-10A cells (Table 2). Both AA and DAH were most prevalent in MCF-7 cells and least present in MCF-10A cells. Of note, AA-derived lipid mediators that promote tumor progression and metastasis, as the cyclooxygenase 2 (COX 2) is an important enzyme of AA cascade.

**Table 1:** Endocannabinoid levels of PEA and OEA determined for breast cancer cells.

Cells	PEA (ng)	OEA (ng)
MCF-10A	0.336	0.061
MCF-7	0.078	0.036
MDA-MB-231	0.117	0.082

Endocannabinoid levels of PEA and OEA determined for breast cancer cells. Values are in ng/10^6^ cells.

**Table 2:** Endocannabinoid levels of Arachidonic acid (AA) and Docosahexaenoic acid (DHA) determined for breast cancer cells.

Cells	AA (ng)	DHA (ng)
MCF-10A	0.668	0.366
MCF-7	2.73	2.65
MDA-MB-231	1.44	1.81

Values are in ng/10^6^ cells.

### Effect of AM11095 on endogenous PEA levels

To evaluate if NAAA inhibition leads to PEA elevation in breast cancer cells in the presence of the AM11095 compound, we examined PEA levels in MDA-MB-231 and MDA-MB-BrM2 cells treated with either vehicle control or with AM11095 (250 nM) for 72 h. AM11095 enhanced endogenous levels of PEA by 1.7–1.9-fold (Table 3).

**Table 3:** Effects of AM11095 on PEA secretion.

Cells	Treatment	PEA fold change from control
MDA-MB-231	Vehicle control	1.0
MDA-MB-231	AM11095	1.8
MDA-MB-BrM2	Vehicle control	1.0
MDA-MB-BrM2	AM11095	1.7

The effect of AM11095 on levels of PEA by incubating MDA-MB-231 cells (10^6^) with 250 nM concentration or vehicle control for 72 h. Cells were harvested and lysed, and PEA levels in cell lysates were measured using LC–MS method. Results are expressed as PEA fold change from control. Values are expressed as means (n = 3).

### AM11095 and PEA mediated effects on cell viability

**Figure 2:**
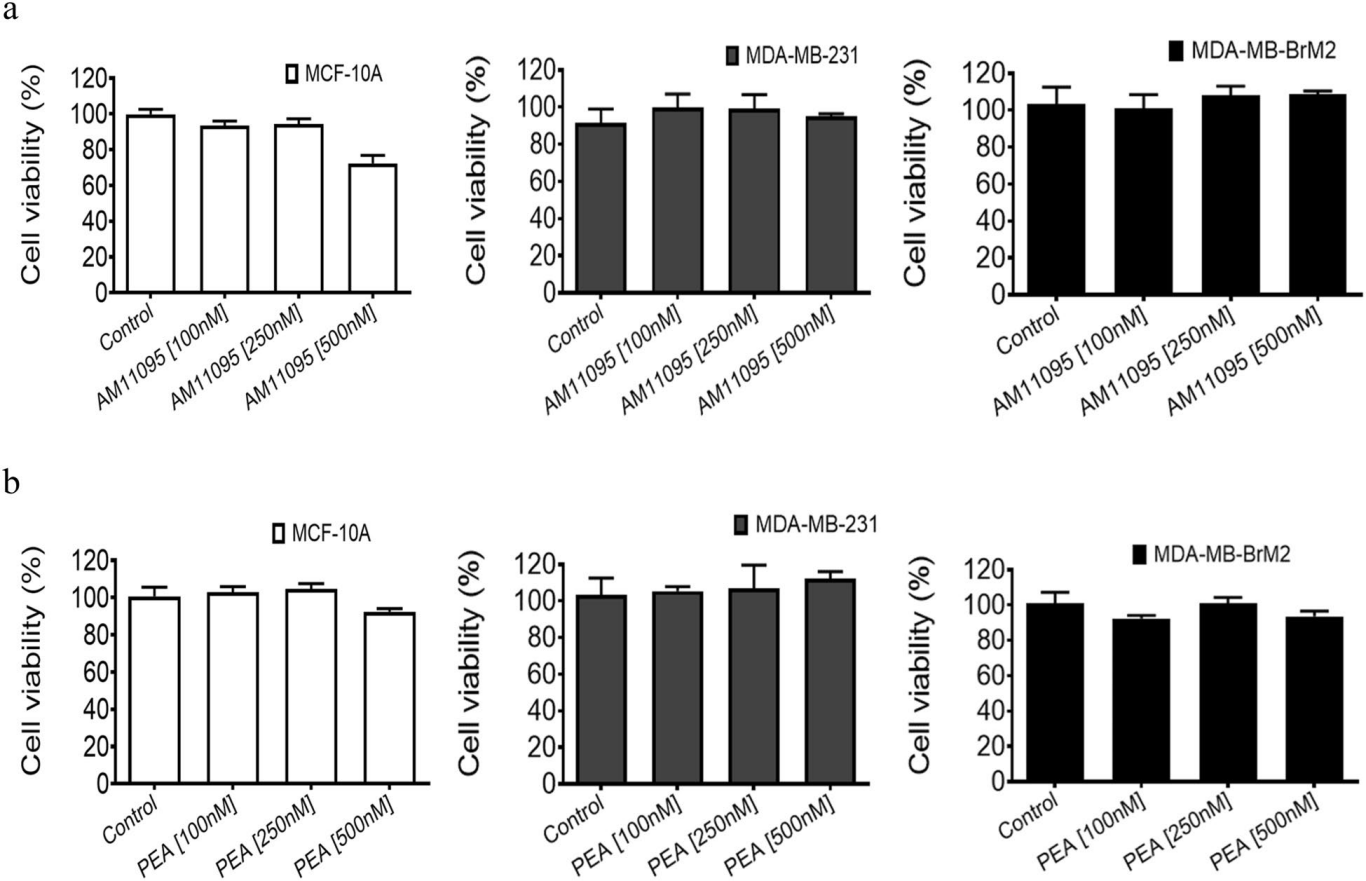
Effect of PEA or AM11095 on cell viability as well as the effect of AM11095 on PEA level in breast cancer cells. (**a**), (**b**) Effects of AM11095 (panel **a**) or PEA (panel **b**) on cell viability on non-tumorigenic and breast cancer cells. Effects of AM11095 (panel **a**) or PEA (panel **b**) on cell viability on non-tumorigenic and breast cancer cells. MCF10A, MCF-7, MDA-MB-231, and MDA-MB-BrM2, 5 × 10^4^ cells/well were incubated for 72 h with AM11095 or PEA at different the concentrations noted in the Figure. Cell viability was assessed using the MTT assay. The percent viability is presented as the means ± S. D (n = 3).

The viability effects and time kinetics of treatment with PEA (Fig. 2b) as compared to AM11095 (Fig. 2a) were performed. The following doses were examined: 100, 250, and 500 nM for 72 h. No significant toxicity on cell viability was observed with PEA or AM11095 on the tested cells (MCF10A, MCF-7, MDA-MB-231, and MDA-MB-BrM2 cells) (Fig. 2).

### Effects of AM11095 on inflammation, activation of NF-kB, and VEGF secretion

First, the effects of AM11095, as compared to vehicle control, on the expression of cytokines/chemokines in breast cancer cells was assessed. Cells were treated with either vehicle control or with AM11095 (250 nM) for 48 h and analyzed by the Milliplex immunobeads panel technology (EMD Millipore’s MILLIPLEX MAP Human Cytokine/Chemokine kit) for the simultaneous quantification of human cytokines and chemokines. AM11095 (Supplemental Table S3), showed some inhibitory effects on the following chemokines/cytokines: IL-4, IL-6, IL-8, INF-alpha2 and G-CSF levels, most noticeable in MDA-MB-231 and MDA-MB231-BrM2 cells. These inflammatory cytokines are all important in tumor—associated inflammation.

**Figure 3:**
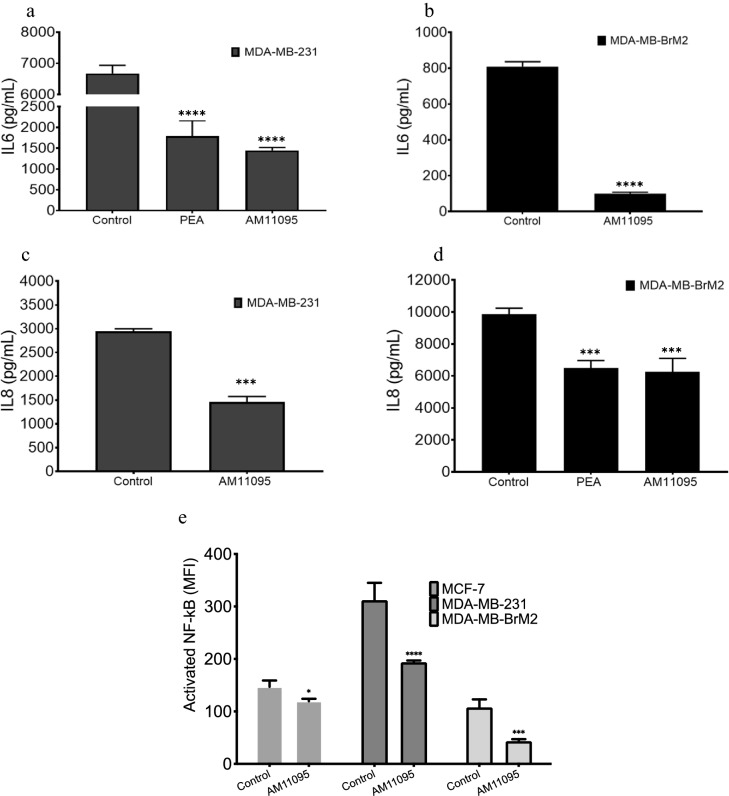
Effects of AM11095 on the expression of chemokines/cytokines. (**a**)–(**d**) Effect of AM10095 on human cytokines/chemokines expression in MDA-MB-231 and MDA-MB-BrM2 cells was assessed using Luminex kit (MILLIPLEX,). Data were adjusted for background, and then the observed concentrations plotted on a log–log scale. Error bars were calculated using the coefficient of variation (%CV) for each sample from all bead fluorescence intensities between 5th centile and 95th centile (trimmed bead %CV). The p-values were calculated by Unpaired Student’s t-Test. (**e**) Effect of NAAA inhibition on the NF-κB activation was assessed by intracellular Bead-based Multiplex Assays using the Luminex technology in cell nuclear samples prepared from breast cancer cells treated with vehicle or AM11095 as indicated.

*Second*, further analysis validated these results that both PEA and AM11095 inhibited both the secretion of IL-6 and IL-8 inflammatory cytokines, which are important in tumor–associated inflammation (Fig. 3a–d).

*Third*, we confirmed these results of PEA and AM11095 as compared to vehicle control on activating cytokines/chemokines in MD-MB-231 cells using human antibody array of cytokines and chemokines. Both IL-6 and IL-8 proteins were inhibited by AM11095 (Supplemental Fig. S1).

*Fourth*, increased of the nuclear factor kappa B (NF-κB) activation levels correlates with poor prognostic outcome and has been implicated in several inflammatory and malignant diseases^37–40^. Since TNBC cells express elevated levels of activated NF-κB, we examined the effects of AM11095 on NF-κB activation using the Milliplex map NF-kB signaling magnetic beads kit 6-plex cell signaling assay (Millipore Sigma, 48-630MAG). Untreated or treated cells, were lysed in Milliplex map lysis buffer containing protease inhibitors. Samples were analyzed according to the assay protocol. The median fluorescence intensity (MFI) was measured with the Luminex system. Inhibition of NF-kB signaling panel was observed in AM11095 treated samples, as compared to control samples, indicating the inhibitory effects of NAAA on NF-kB pathway (Fig. 3e).

**Figure 4:**
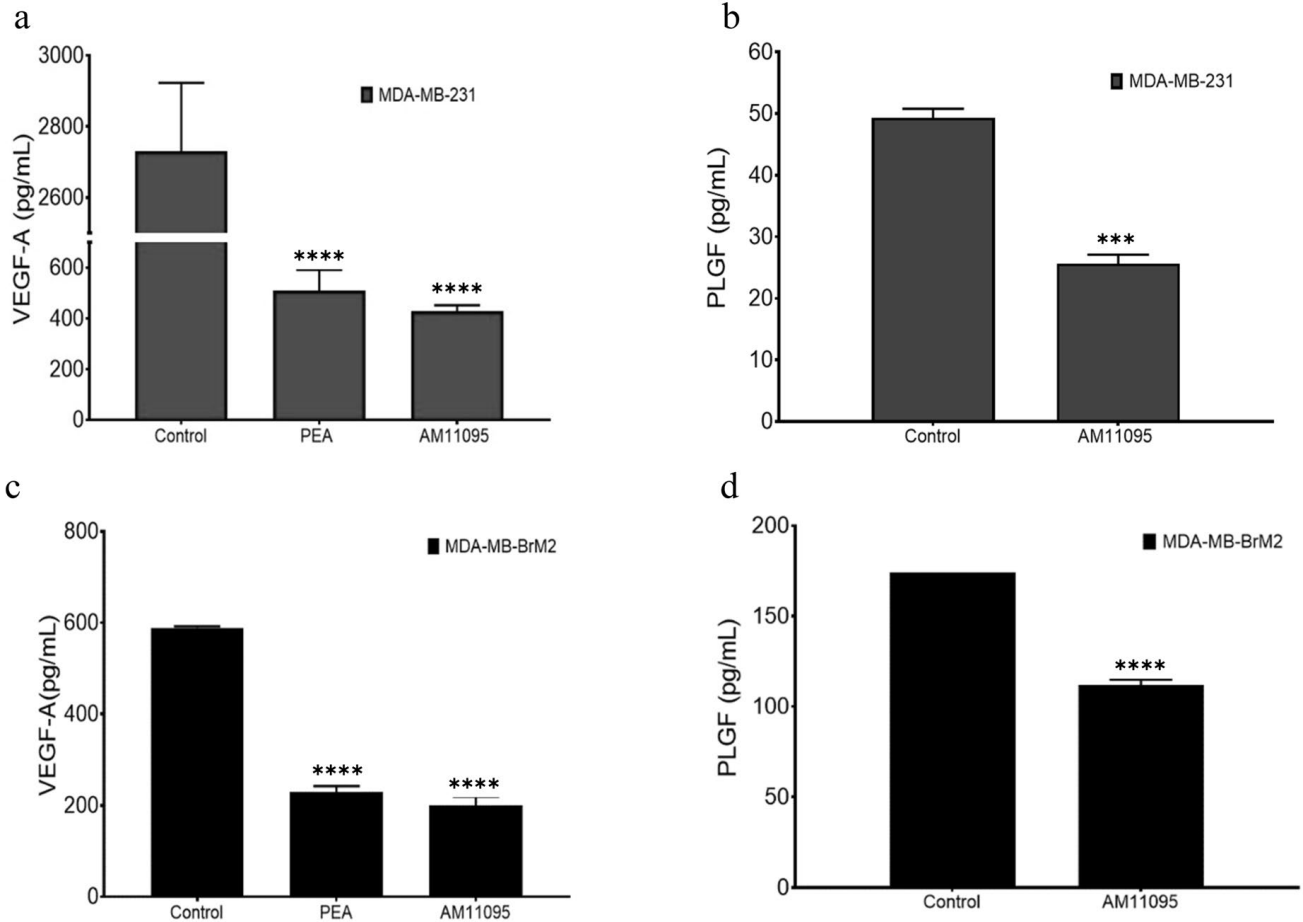
(**a**)–(**d**) Angiogenesis Bead-Based Multiplex Assays: The levels of the angiogenesis factors in culture supernatants of MDA-MB-231 (panels: **a**, **b**) and MDA-MB-BrM2 (panels: **c**, **d**) cells with notable differences between AM11095-treated samples vs. vehicle control -treated samples. Each factor is listed on the Y-axis of the corresponding graph. The concentrations were assayed with Luminex kit (MILLIPLEX). Data were adjusted for background, and then the observed concentrations were plotted on a log–log scale. Error bars were calculated using the coefficient of variation (%CV) for each sample from all bead fluorescence intensities between 5th centile and 95th centile (trimmed bead %CV). The p-values were calculated by Unpaired Student’s t-Test.

*Fifth*, we performed Angiogenesis Bead-Based Multiplex Assays, which enable the simultaneous analysis of multiple angiogenic biomarkers. As shown in Fig. 4, we observed significant inhibitory effects of AM11095 on the secretion levels of VEGF and PLGF from both MDA-MB-231 and MDA-MB-BrM2 cells.

### Effects of AM11095 on cell invasion and migration in vitro

**Figure 5:**
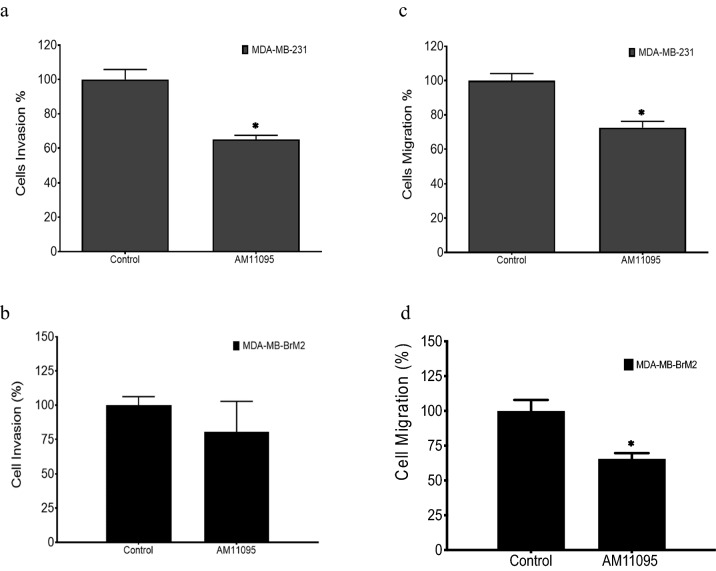
Effects of AM11095 on invasion, and migration of TNBC cells. (**a**), (**b**) Cells were either untreated or treated with either vehicle control or with AM11095 as indicated for 72 h and then assayed for cell invasion. Data shown are representative of at least three independent assays performed on duplicate or triplicate wells. The error bars indicate standard deviations. (**c**), (**d**) Cells were seeded over the monolayer in the presence or absence of AM11095 for 72 h. The resulted fluorescence from Calcein AM cleavage by the migrated cells in bottom plate to free Calcein was measured using a Biotek reader instrument and expressed as the percentage of cells migrated relative to control. Data shown are representative of at least three independent assays performed on duplicate or triplicate wells. The error bars indicate standard deviations. The p-values were calculated by Unpaired Student’s t-Test. (*) *P* ≤ 0.05, (**) *P* ≤ 0.01, (***) *P* ≤ 0.001, (****) *P* ≤ 0.0001, (n.s) not significant.

The effects of AM11095 on tumor cell invasion and migration were examined. For invasion assay, treated and untreated cells (MDA-MB-231 and MDA-MB-BrM2 cells) were added to the top chamber and allowed to migrate through the filter membrane coated with Basement Membrane Extract (BME), which mimics the extracellular matrix, to the bottom chamber for 72 h. As shown in Fig. 5 (panels a, b), inhibition of tumor invasion by AM11095 was observed in MDA-MB-231 cells, but not in MDA-MB-BrM2 cells. Further, the effects of AM11095 on tumor cell migration was examined and about 30% inhibition of tumor cell migration was observed in the presence of AM11095 in both cell lines (Fig. 5c, d).

### Effects of AM11095 on tumor growth and progression in vivo

To monitor tumor growth in vivo and the effects of AM11095 on tumor growth, we employed MRI method which combines the ability to obtain high resolution MRI data^36^. The presence of the tumor cells causes a distortion in the magnetic field and leads to abnormal signal hypo-intensities in tumor images. As tumors form, changes to the tissue result in the tumor appearing brighter than the surrounding tissue in MRI. These cellular MRI techniques compare the growth of the tumor cells following different treatments and conditions. The tumor TNBC cells are tagged with Thymidine-GFP-Luciferase (TGL) gene markers. To examine the in vivo effects of AM11095 on tumor growth spreading, GFP-MDA-MB-BrM2 cells were mixed with Matrigel to form a scaffold to support the implantation of tumor cells in vivo (n = 10 per group per treatment). These mixtures were administered (5 × 10^4^ cells) intraperitoneally (IP) in Nude mice. Mice were then injected with either untreated or treated with vehicle control or with AM11095 twice a week (at 30 mg/Kg). Untreated mice and vehicle control treated mice developed tumors at day 28 post injection (Endpoint of the experiment). Areas containing tumor cells appear as regions of low intensity on MR images, creating negative contrast. As tumor cells progress to other organs and form metastases, there are changes to the tissue which result in the tumor appearing brighter than the surrounding tissue in MRI. These cellular MRI techniques compare the growth of the tumor cells following different treatments and conditions.

**Figure 6:**
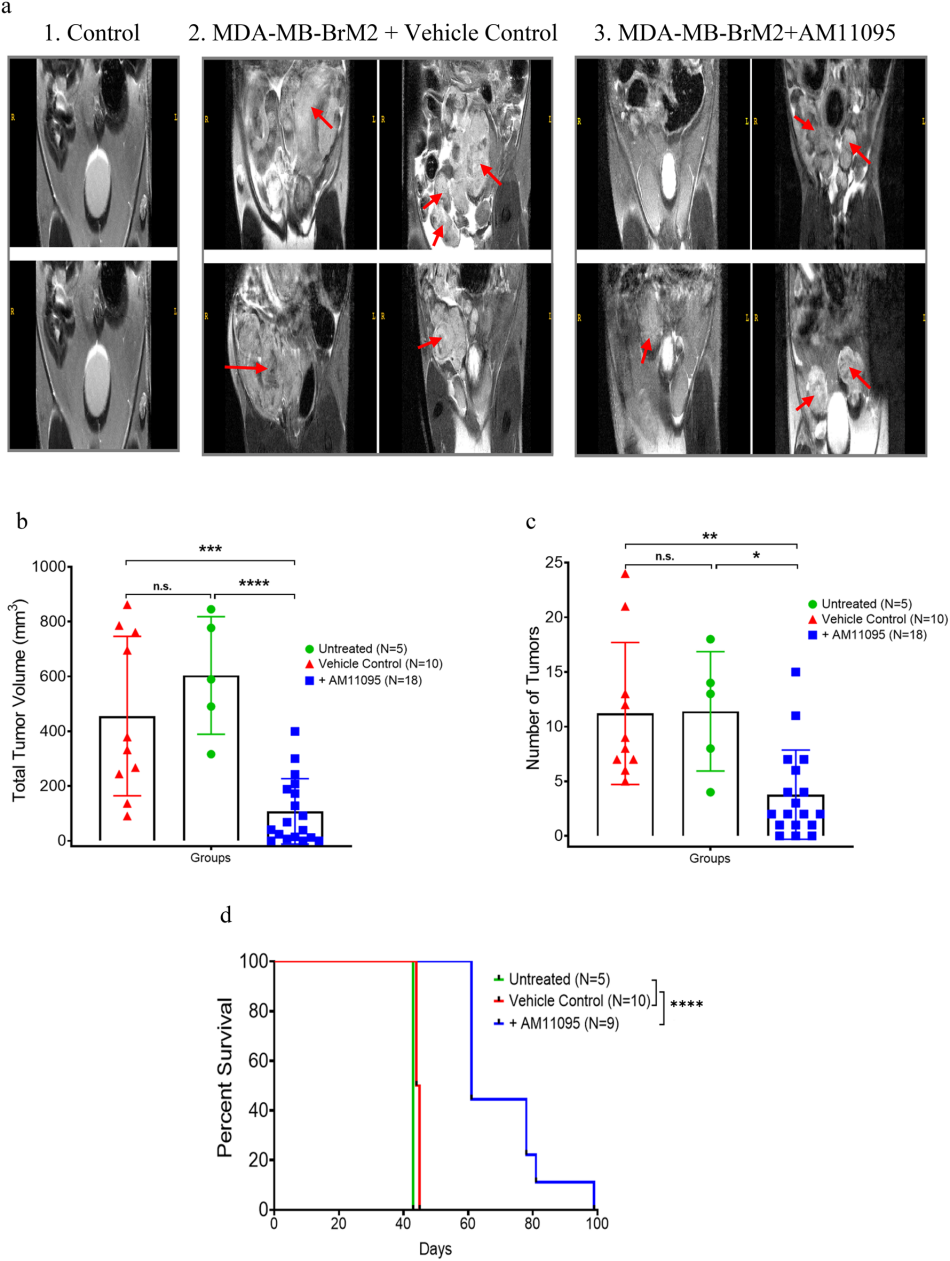
MRI analysis and Endpoint visualization of control nude mice and nude mice administered with tumor cells and either untreated, or vehicle control treated or AM11095 treated at day 28 endpoint. (**a**) (1) Representative images at day 28 showing visualization of control nude nice. (2) Representative images at day 28 showing regions of signal hypersensitivity where tumors developed in nude mice administered with tumor cells and treated with vehicle control. (3) Representative images at day 28 showing regions of signal hypersensitivity where tumors developed in nude mice administered with tumor cells and treated with AM11095. Arrows show the tumor areas. (**b**) The volume size of tumors in the nude mice were analyzed at endpoint in mice administered with tumor cells and either untreated or treated with vehicle control or with AM11095. Each symbol represents the value of an individual mouse. The p-values were calculated by Unpaired Student’s t-Test. (**c**) Number of tumors detected with MRI (n = 20) at endpoint in mice administered with tumor cells and either untreated or treated with vehicle control or with AM11095. Each symbol represents the value of an individual mouse. The *p* values were calculated by Unpaired Student’s t-Test. (**d**) Survival analysis of AM11095 treated mice. Mice were divided into three groups based on the treatment. The p-values were calculated by Unpaired Student’s t-Test.

As shown in Fig. 6, representative images at day 28 images for control healthy untreated mice, mice treated with tumor cells + vehicle control and mice treated with tumor cells + AM11095 are shown. MRI body images revealed that the average numbers and volume of tumors was significantly higher in mice treated with tumors + vehicle control (Fig. 6) and significant tumor burden was also observed in other tissues such as lung (Fig. 6). The tumors grew more rapidly in these mice and as tumor cells progress to other organs and form metastases, there were changes to the tissues which result in the tumor appearing brighter than the surrounding tissue in MRI. Interestingly, body images revealed that the average numbers and volume of tumors was significantly reduced following treatment with AM11095 (Fig. 6a). The MRI images revealed the mean volume in untreated tumors and vehicle treated groups were twofold and threefold increase respectively, as compared to the mean in nude mice treated with AM11095 (Fig. 6b). Further, the mean number of tumors in untreated mice and vehicle control treated mice were approximately 2 times greater than in nude mice treated with AM11095 (Fig. 6c). On day 28, the mean number of tumors per mouse was significantly lower and no tumor burden was observed following treatment with AM11095 (Fig. 6c). Importantly, while there was no difference in survival between the two groups of mice (untreated and vehicle control), an increase in the survival of mice were observed when mice were treated with AM11095 (Fig. 6d). Thus, AM11095 may have a survival benefit in mice in addition to its inhibiting tumor growth in vivo.

Tumor sections analysis, hematoxylin–eosin (H&E) staining and immunostaining: In the untreated tumors, there was extensive (~ 60%) necrosis within the mass, particularly within central regions of cell growth (Supplemental Fig. S2-B). The tumors were moderately heterogeneous with poorly differentiated individual cells, cell clusters, and spindloid components within a thin fibrous network (Supplemental Fig. S2-C, representative images). Some borders of the tumors were well demarcated but with regional invasion of the adjacent adipose tissue and lymphatic vessels (Supplemental Fig. S2-D and 2-E, representative images). Individual tumor cells were pleomorphic exhibiting moderate anisocytosis and anisokaryosis, occasional large pleomorphic nuclei, and dark basophilic stippled chromatin with occasional giant cells present. There were frequent mitoses, and occasional bizarre mitotic figures (Supplemental Figure S2-F, representative images). There was also a mild mixed (mostly neutrophilic) inflammatory infiltrate within the tumor stroma and adjacent tissues (Supplemental Fig. S2-E). In the AM11095 treated tumors, there was extensive (~ 50%) necrosis within the mass, and the tumors were moderately heterogeneous with poorly differentiated individual cells, cell clusters, and spindloid components within a thin fibrous network (Supplemental Fig. S3-C, representative images). Interestingly, there was no clear invasion of tumor cells beyond the surface of the tissue. The Individual tumor cells were pleomorphic exhibiting moderate anisocytosis and anisokaryosis, occasional large pleomorphic nuclei, and dark basophilic stippled chromatin with occasional giant cells and signet ring-like cells present (Supplemental Fig. S3-D). Interestingly, there was less infiltrated immune cells (mostly neutrophilic), inflammatory infiltrate within the tumor stroma and adjacent tissues. However, immunohistochemistry analysis of tumor cells with specific antibodies for IL-6 and VEGF revealed few positive immunostainings of these two targets (Fig. S4 and Table S4), and there were no statistical differences between the two groups, untreated tumors and AM11095 treated tumors.

Together, these studies showed that mice administered with human MDA-MB-BrM2 cells treated with AM11095 had significant decrease of tumor numbers with lower volume of tumors and increased mice survival, as compared to mice untreated or treated with vehicle control.

## Discussion

Patients with TNBC and/or metastatic breast cancer start their treatment with heavy tumor burden, resulting in high numbers of tumor cells that have acquired drug resistance. Unfortunately, TNBC recurrence is associated with late stage of the disease and has poor prognosis with noticeably brief period of recurrence. Therefore, developing treatments that can decrease tumor growth, prolong remission, and decrease chemo resistant tumor cells, is especially important in developing new treatments. The rational of this study was to delineate the role of PEA/NAAA pathway in modulating lipid metabolism and decreasing the acquisition of aggressive TNBC phenotype. Thus, we investigated TNBC-associated inflammation by inhibiting NAAA enzyme, leading to increase in PEA lipid levels.

In this study, we provide data that NAAA isoforms are highly expressed in breast cancer cells as compared to normal epithelial cells. Four major splice variants in MCF-7, MDA-MB-231 and MDA-MB-BrM2 cells, which were composed of exons 1–11, exons 1–10 and 12, exons 1–9 and 12, and exons 1–8 and 12, respectively (a1, a2, b2, and c2)^35^ were found. We then performed quantitative polymerase chain reaction methods to individually quantify these NAAA variants as well as collectively measure all the variants. The total levels of NAAA mRNAs in breast cancer cells were significantly higher as compared to normal breast epithelial cells MCF-10A cells. Variants a1 and a2 encoded the same full-length NAAA protein, which were catalytically active, while b2 and c2 were translated to C-terminally truncated proteins were catalytically inactive precursors, as reported previously^35^. As shown in Fig. 1, breast cancer cells express significant levels of A2, B2, C2 while normal MCF-10A cells has exceptionally low expression of these isoforms. The high expression of NAAA splice isoforms in breast cancer cells were reduced in cells treated with siRNA NAAA (Fig. 1c, d) indicating the specificity of these Naaa variant analysis using this approach^35^. These data suggest that NAAA splice variants might be used as biomarkers for TNBC progression. Future studies will examine the PEA hydrolytic activity of NAAA splice variants found in TNBC cells.

Lipids play important roles in metastasis and cancer progression^21^. Recently, breast cancers capable of metastasis formation to the brain showed evidence of altered lipid metabolism and perturbation of lipid metabolism in tumor cells inhibited brain metastasis development^53,54^. Lipids have many pleiotropic effects and their functions in inflammation and cancer depend on concentration and on context. Here we show that TNBC cells express the N-acyl ethanolamides, PEA and OEA and elevated levels of the two fatty acid cores AA and DHA, as determined by LC-Atmospheric Pressure Chemical Ionization-MS. Further, TNBC cells express high levels of NAAA is also involved in TNBC associated inflammation and tumor growth.

AM11095 targets the biolipid PEA, which has anti-inflammatory and/or analgesic properties. Treatment of tumor cells with AM11095 led to an elevation of PEA (Table 3). Endogenous molecules, choline-or ethanolamine-containing phospholipids and dihydrolipoic acid were reported to potently stimulate NAAA as substitutes for polyoxyethylene p–t-octylphenyl ether-type detergent and DTT, respectively, contributing to keeping NAAA active in lysosomes^49^. Since preferential hydrolysis of PEA over other NAEs was found with these stimulators and since there is a compensatory increase in the expression level of NAAA that was not observed in FAAH knockout mice^49^, we aim to will determine the effects of AM11095 on the expression levels of NAEs in future studies.

Inhibition of PEA’s catalytic enzyme NAAA via AM11095 led to inhibition of inflammatory soluble mediators in TNBCs and reduced NF-kB activation. Chronic secretion and exposure to IL-6 proinflammatory cytokine plays a significant role in tumor promoting inflammation resulting in a feedback loop of persistent activation of pro-inflammatory signaling and tumor progression^44^. Prolonged exposure of tumor cells to Il-6 or IL-8 induced the appearance of senescent cells and together with the inflammatory milieu, reinforce the tumorigenic capabilities of the tumor cells and increasing tumor aggressiveness^44^. A major common pathway to most cancers is the activation of the nuclear factor kappa B (NF-κB)^6,11,12,27^. NF-κB is a transcription factor that links inflammatory gene(s) activation and gene controlling cell growth factors, angiogenesis, and chemokine/cytokine regulation. Our results showed that TNBC tumor cells secreted elevated levels of proinflammatory markers including IL-6, IL-8, VEGF, and higher NF-κB transcription factor activation as compared to normal breast epithelial cells. These results were validated by repeated Milliplex assays and proteome profiler human cytokine array kit (Supplemental Fig. S1 + Table S3). The IL-6 cytokine signaling molecule drives chronic inflammation by inducing the activation of the NF-κΒ cell nuclear transcription factor pathway. The NF-κΒ activated pathway mediates tumor induced inflammation by transcribing chemokine/cytokine such as IL-8, which in turn serves to promote tumor progression leading to a feedforward pro-tumor inflammation. Thus, inhibition the pro-inflammatory factors such as IL-6 and IL-8 should lead to decrease in NF-κB activation and block the pro-inflammatory cytokine secretion. These findings can translate to anti-inflammatory therapies in TNBCs.

Interestingly, we also observed a significant reduction of VEGF and PLGF angiogenic factor secretion with the AM11095 or PEA treatment (Fig. 4). This finding correlates with the need of these aggressive cells for nutrients and oxygen associated with the increased metabolism and proliferation of these cells. Pro-angiogenic responses have been associated with increased VEGF, IL-6, and IL-8 expression, in accordance with our results.

There are multiple imaging protocols for detecting the gross lesions that results from tumor growth. Based on our knowledge, this is the first report of using MRI^36^ to detect tumor growth in mice and to monitor these tumors following treatment. The high-resolution cellular MRI allowed us to characterize the MDA-MB-BrM2 cells in nude mouse model. Of note, the MDA-MB-BrM2 cell line was developed to selectively grow in distant sites in mice to form metastases especially in the bone, lungs, and brain. We found differences in tumor incidence, volumes, and body tumor burden between the untreated mice and vehicle treated mice as compared to AM11095 treated mice (Fig. 6). Specifically, the in vivo studies showed differences in tumor progression in Nude mice following AM11095 treatment compared to untreated mice. The size of the signal void created by the tumor cells was much larger than the signals in the AM11095 treated mice. In the controlled untreated mice, the tumors developed more quickly with substantial body tumor burden in various organs. Exploratory mouse body MRI in these two groups of mice revealed additional tumor burden due to tumors in other tissues. These tumors were mostly absent in the AM11095 treated mice. The limitation of these in vivo studies is the lack of luminescent/fluorescent imaging to gain access on the metastasis spreading. Since NAAA knockout mice are not available, future studies in my lab are aimed to genetically validate the importance of NAAA for tumor growth and metastatic potential, by employing shRNAs to knockdown NAAA, using pLKO lentiviral vectors. Using this mice model, we will aim to show that the observed effects of AM11095 in vivo are not due to off-target effects and to further demonstrate the specificity of AM11095 mediated effects in vivo.

Interestingly, several studies reported recently new compounds that inhibit NAAA activities. Carmofur, a 5-fluorouracil-releasing drug and clinically used as a chemotherapeutic agent, attenuated LPS-induced acute lung injury^50^ via inhibition of FAAH and Naaa^50^. Another study showed that inhibition of NAAA by ARN16186, a small molecule that specifically designed and synthesized by Dr. Reggiani’s lab^51^ was shown to reduce T-cell infiltration in a mouse model of multiple sclerosis. Furthermore, Studies by Dr. Li’s lab^52^ showed that F215, a NAAA inhibitor, attenuated osteoarthritis^52^. Additional study by Dr. Li’s lab^55^ showed that a new NAAA inhibitor F96 attenuated blood brain barrier disruption and secondary injury after traumatic brain injury. Together, these studies strongly suggest that NAAA is a new important target for treatment of inflammation associated with brain injury, lung injury and several other diseases.

In summary, we conclude that NAAA is important in TNBC- associated inflammation and tumor growth in vivo. These results should provide a better understanding of the impact of targeting TNBC tumor growth by lipid modulation via specific inhibitors to NAAA.

## Supplementary Information

Supplementary Information.

## Data Availability

All data generated and/or analyzed during this study are included in this published article. Additional data are provided in Supplemental Information 1.

## Abbreviations

2-AG: 2-Arachidonoylglycerol
AA: Arachidonic acid
AEA: N-arachidonoyl-ethanolamine
CB_1_: Cannabinoid receptor type 1
CB_2_: Cannabinoid receptor type 2
DHA: Docosahexaenoic acid
ECM: Extracellular matrix
EGF: Epidermal growth factor
FFAs: Free fatty acids
IHC: Immunohistochemistry
IL: Interleukin
MFI: Median fluorescence intensity
NAAA: N-acylethanolamine acid amidase
NF-kB: Nuclear factor kappa-light-chain-enhancer of activated B cells
OEA: Oleoyl ethanolamide
PEA: Palmitoylethanolamide
PPARs: Peroxisome proliferator-activated receptors
RT-PCR: Reverse transcription polymerase chain reaction
TGL: Thymidine kinase (T), GFP (G), and luciferase (L)
TME: Tumor microenvironment
TNBC: Triple negative breast cancer
VEGF: Vascular endothelial growth factor
WB: Western blotting
