# Supplementary material for: Inhibition of triple negative breast cancer-associated inflammation and progression by N- acylethanolamine acid amide hydrolase (NAAA)
Source: Sci Rep. 2022 Dec 23;12:22255. doi: 10.1038/s41598-022-26564-6 (PMC9789040; doi:10.1038/s41598-022-26564-6)
Supplement: Supplementary file 1 — Supplementary Information. [file 41598_2022_26564_MOESM1_ESM.docx]

| **Oligo Name** | **Exon(s)** | **Sequence (5' to 3')** |
| --- | --- | --- |
| **Forward primers** | | |
| f1 | 6 | 5'-GCTGATGTTTATTACATTGTTGGTGGCACGTC-3' |
| f5 | 8 | 5'-CAAACCTCAGCCTGGAGGC-3' |
| f7 | 8 | 5'-CAAGCAAACCTCAGCCTGGAGGC-3' |
| f11 | 1 | 5'-GCGGAAGCTTGAGCCCGAGCCATGCGGAC-3' |
| **Reverse primers** | | |
| r2 | 8 | 5'-GAAAAGTGCCTCCAGGCTGAGGTTTG-3' |
| r3 | 11 | 5'-TCACTTTGTCTTATTTTTTAAGGTGCAGCTCTTCAAG-3' |
| r4 | 12 | 5'-CTGACAATCACCTGATGGGTTCTTCTTGCTC-3' |
| r6 | 10, 11 | 5'-CAGCTCTTCAAGAATTTCATTTTTTAAAAAATCATCTTTC-3' |
| r8 | 10, 12 | 5'-CAATCACCTGATGGGTTCTTCTTGCTCACTTT-3' |
| r9 | 9, 12 | 5'-CAATCACCTGATGGGTTCTTCTTGCTCATTGT-3' |
| r10 | 8, 12 | 5'-CCTGATGGGTTCTTCTTGCTCACTGG-3' |
| r12 | 9, 12 | 5'-GAGGGCCCTCGAGTCACCTGATGGGTTCTTCTTGCTCATTGTTATAAACTGGAACCACCGACA-3' |
| r13 | 8, 12 | 5'-GAGGGCCCTCGAGTCACTGGAAAAGTGCCTCCAGGCTGAGG-3' |

**Table S1**

**Table S1: NAAA PCR primers sequences (as published by Y. Sakura et al., Biochimica et Biophysica Acta 1861 (2016) 1951–1958.**

**Table S2: ON-TARGETplus Human NAAA (27163) siRNA – SMARTpool (L008297-01-0005) gene sequence**

ON-TARGETplus SMARTpool siRNA J-008297-09, NAAA

**Target Sequence:** GCACGUGUUAAUCGGAAAA

Mol. Wt. Ext. Coeff.

13,415.1 (g/mol) 374,423 (L/mol·cm)

ON-TARGETplus SMARTpool siRNA J-008297-10, NAAA

**Target Sequence:** UUGGGAAUGUCUUACGCAA

Mol. Wt. Ext. Coeff.

13,415.0 (g/mol) 373,711 (L/mol·cm)

ON-TARGETplus SMARTpool siRNA J-008297-11, NAAA

**Target Sequence:** CAUUUACCAUGGUCGGAAU

Mol. Wt. Ext. Coeff.

13,415.1 (g/mol) 377,627 (L/mol·cm)

ON-TARGETplus SMARTpool siRNA J-008297-12, NAAA

**Target Sequence:** GGGUGGUCAUCACGAGGAA

Mol. Wt. Ext. Coeff.

13,460.1 (g/mol) 362,675 (L/mol·cm)

**Table S3**

| **Chemokines/**  **cytokines** | **Condition** | **MCF-10A** | | | **MCF-7** | | | **MDA-MB-231** | | | **MDA-MB-BrM2** | | |
| --- | --- | --- | --- | --- | --- | --- | --- | --- | --- | --- | --- | --- | --- |
|  |  | **Amount (pg/mL)** | **S.D** | **p-value** | **Amount (pg/mL)** | **S.D** | **p-value** | **Amount (pg/mL)** | **S.D** | **p-value** | **Amount (pg/mL)** | **S.D** | **p-value** |
| **IL-6** | Control | 283.7 | 12.5 | p<0.0001 | 33.7 | 0.6 | p<0.0001 | 6675.0 | 263.0 | p<0.0001 | 809.0 | 27.0 | p<0.0001 |
|  | AM11095 | 36.0 | 1.0 |  | 3.0 | 0.0 |  | 1445.3 | 73.5 |  | 100.7 | 8.5 |  |
| **IL-8** | Control | 813.3 | 46.0 | p=0.0005 | 0.0 | 0.0 | N/A | 2948.3 | 51.5 | p<0.0001 | 9864.3 | 379.1 | P=0.0025 |
|  | AM11095 | 534.3 | 9.5 |  | 0.0 | 0.0 |  | 1463.3 | 110.0 |  | 6269.0 | 836.8 |  |
| **G-CSF** | Control | 6726.3 | 325.0 | p=0.0001 | 26.0 | 2.0 | p=0.0002 | 3202.7 | 107.0 | p<0.0001 | 16057.0 | 0.0 | P=0.0336 |
|  | AM11095 | 3379.0 | 191.0 |  | 10.3 | 0.6 |  | 445.7 | 28.5 |  | 12838.3 | 1754.4 |  |
| **INF-Alpha-2** | Control | 6.3 | 0.6 | p=0.0161 | 4.7 | 1.5 | p=0.0061 | 8.7 | 0.6 | p=0.0031 | 19.3 | 1.5 | p=0.0002 |
|  | AM11095 | 5.0 | 0.0 |  | 0.0 | 0.0 |  | 5.7 | 0.6 |  | 7.0 | 0.0 |  |
| **IL-4** | Control | 0.0 | 0.0 | N/A | 0.0 | 0.0 | N/A | 4.3 | 0.6 | p=0.0002 | 4.7 | 0.6 | p=0.0075 |
|  | AM11095 | 0.0 | 0.0 |  | 0.0 | 0.0 |  | 0.0 | 0.0 |  | 3.0 | 0.0 |  |

**Figure S3: Effects of AM11095 on chemokines/cytokines release.**

Effect of AM10095 on human cytokines/chemokines release in MCF10A, MCF-7, MDA-MB-231, and MDA-MB-BrM2 cells was assessed using Luminex kit (MILLIPLEX,). Data were adjusted for background, and then the observed concentrations plotted on a log–log scale. Error bars were calculated using the coefficient of variation (%CV) for each sample from all bead fluorescence intensities between 5th centile and 95th centile (trimmed bead %CV). The p-values were calculated by Unpaired Student’s t-Test. (✱) P ≤ 0.05, (✱✱) P ≤ 0.01, (✱✱✱) P ≤ 0.001, (✱✱✱✱) P ≤ 0.0001, (n.s) not significant.

**Figure S2**

a. (1) (2) b.

**
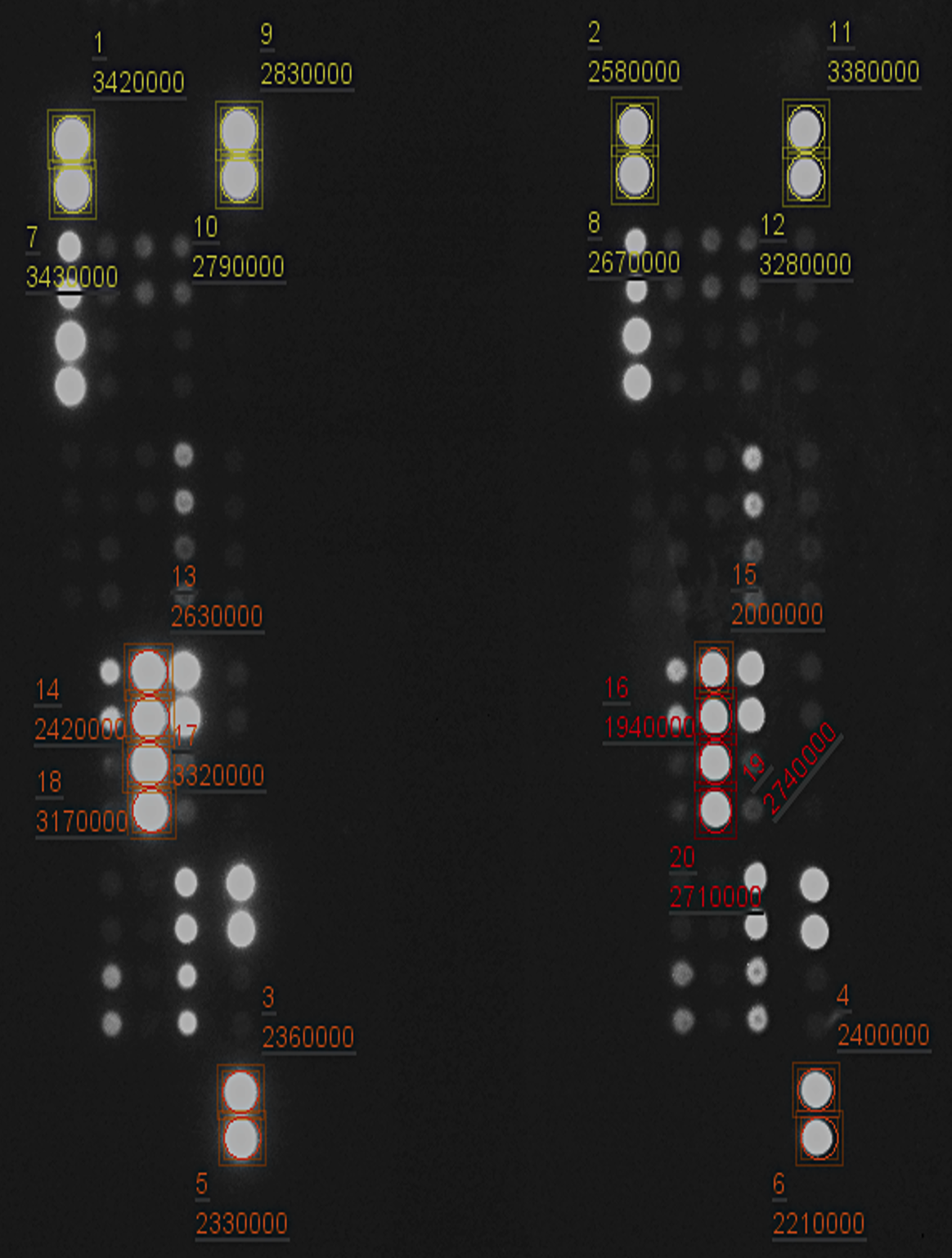
**

**﹡**

**﹡**

2

1

**Figure S2: Human cytokine expression array in MDA-MB-231**

**a (1):** Represents cytokines secretion in untreated MDA-MB-231

**a (2):** Represents cytokines secretion in AM11095 treated MDA-MB-231

**b:** Represents the quantitation of IL-6 and IL-8 in treated and untreated MDA-MB-231

The p-values were calculated by Unpaired Student’s t-Test. (✱) P ≤ 0.05, (✱✱) P ≤ 0.01, (✱✱✱) P ≤ 0.001, (✱✱✱✱) P ≤ 0.0001, (n.s) not significant.

**Figure S3**


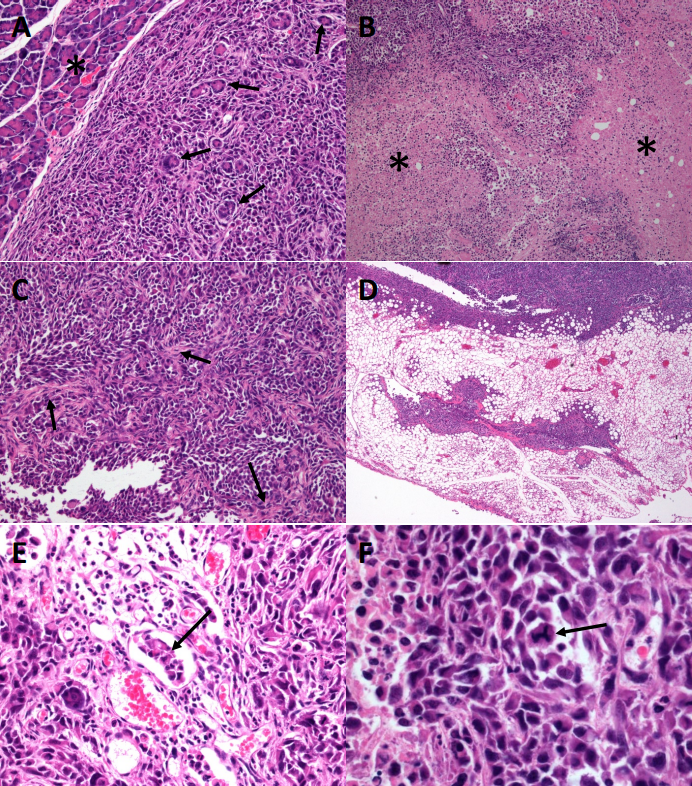


**Figure S3: Tumors in untreated control mice Hematoxylin-eosin immunohistochemistry results.**

A) Normal tissue (*) and remnant acini (arrows) at the periphery of the tumor. 200X. B) Extensive necrosis is present (*). 100X. C) Cells are poorly differentiated with spindloid components and a thin fibrous network (arrows). 200X. D) Regional invasion of the adjacent adipose tissue (40X) and, E) lymphatic vessels (arrow). 400X. F) Frequent mitoses and occasional bizarre mitotic figures (arrow). 800X.

**Figure S4**


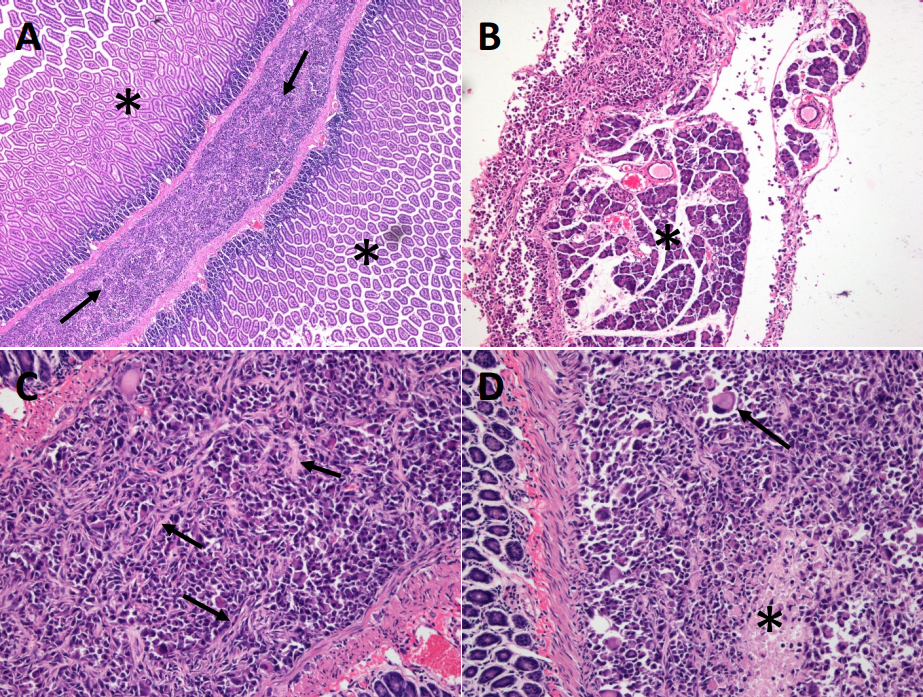


**Figure S4:** AM11095 tumor treated mice Hematoxylin-eosin immunohistochemistry results.

A) Normal tissue (*) with tumor cells effacing serosa (arrows). 40X. B) Infiltration into surrounding mesentery and pancreas (*). 100X. C) Cells are poorly differentiated with spindloid components and a thin fibrous network (arrows). 200X. D) Necrosis (*) and occasional giant cells and signet ring-like cells present (arrow). 200X.

**Figure S5:**

IL-6


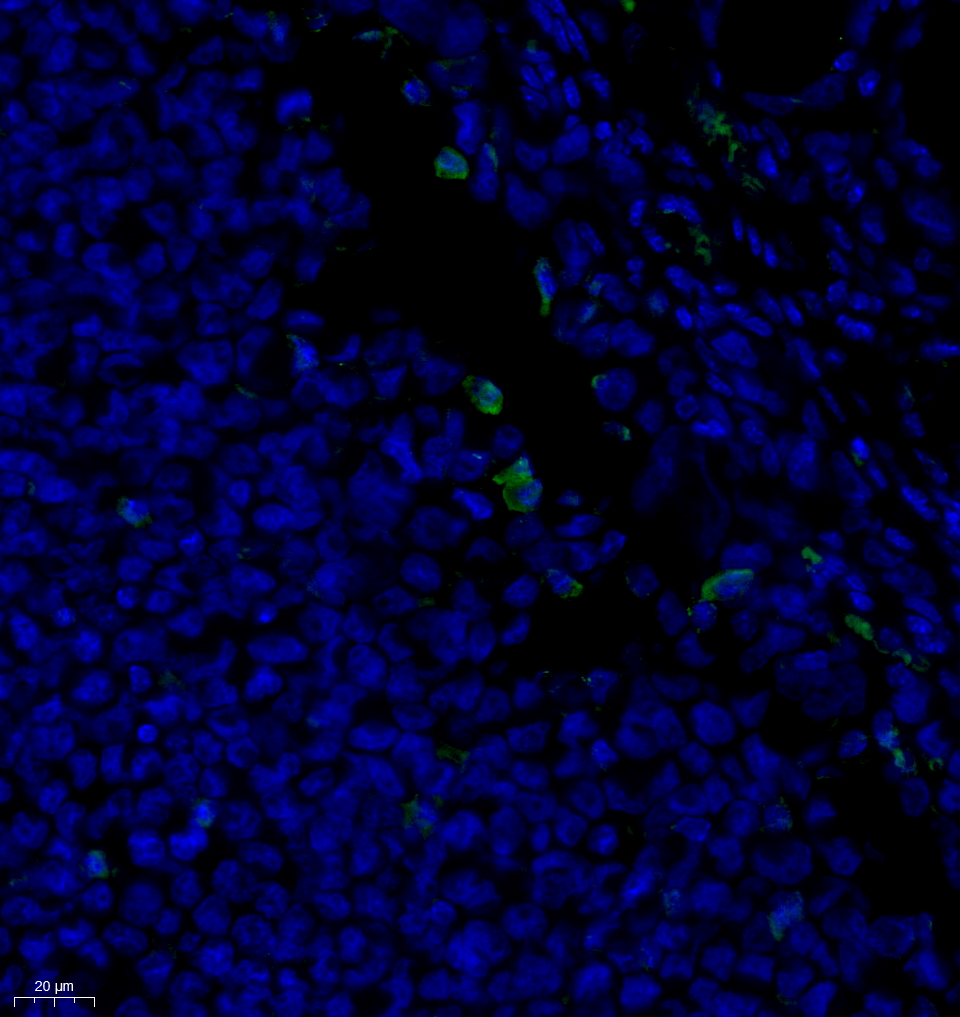

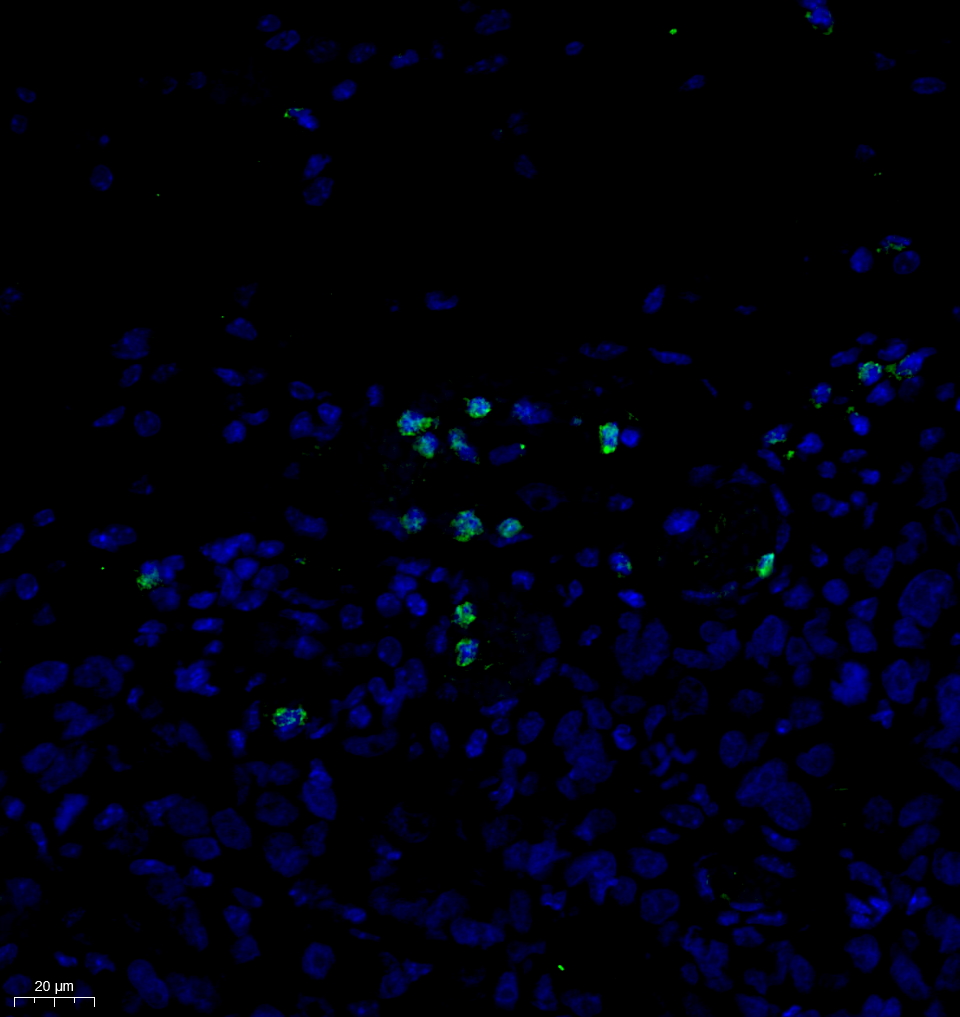

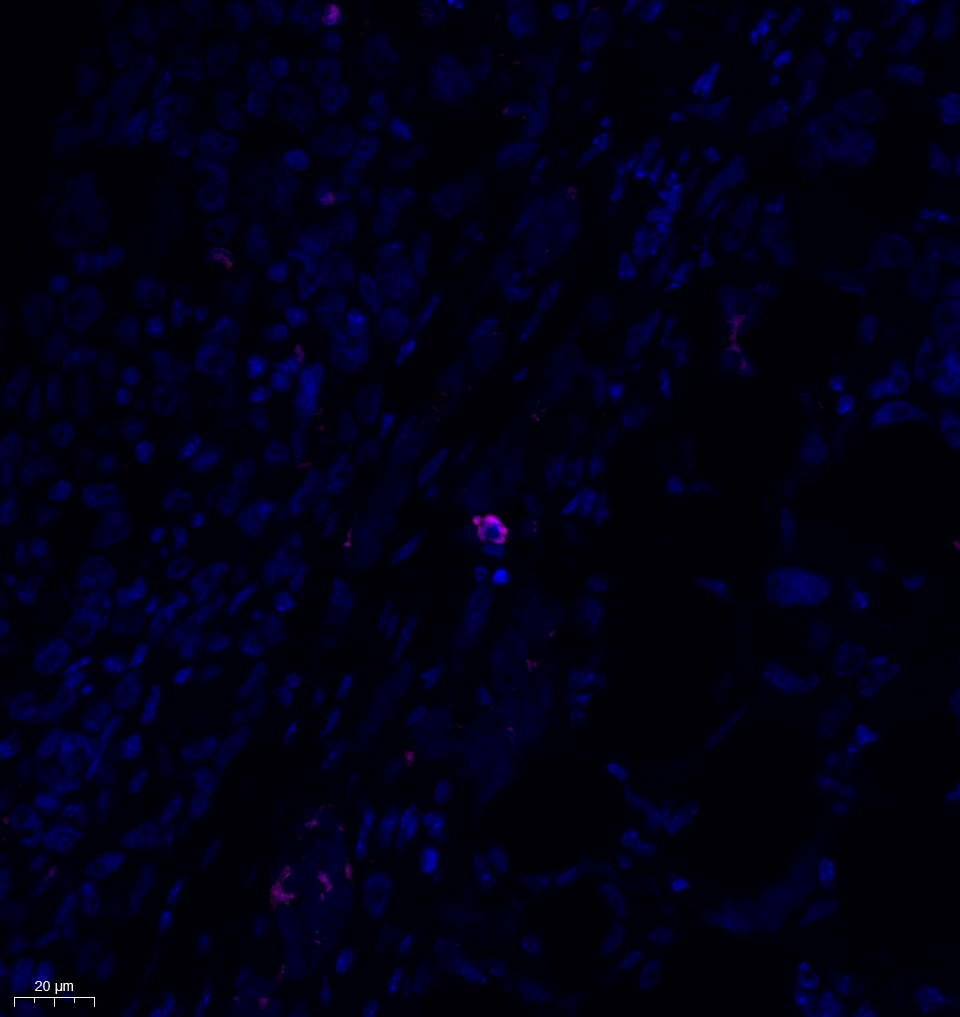

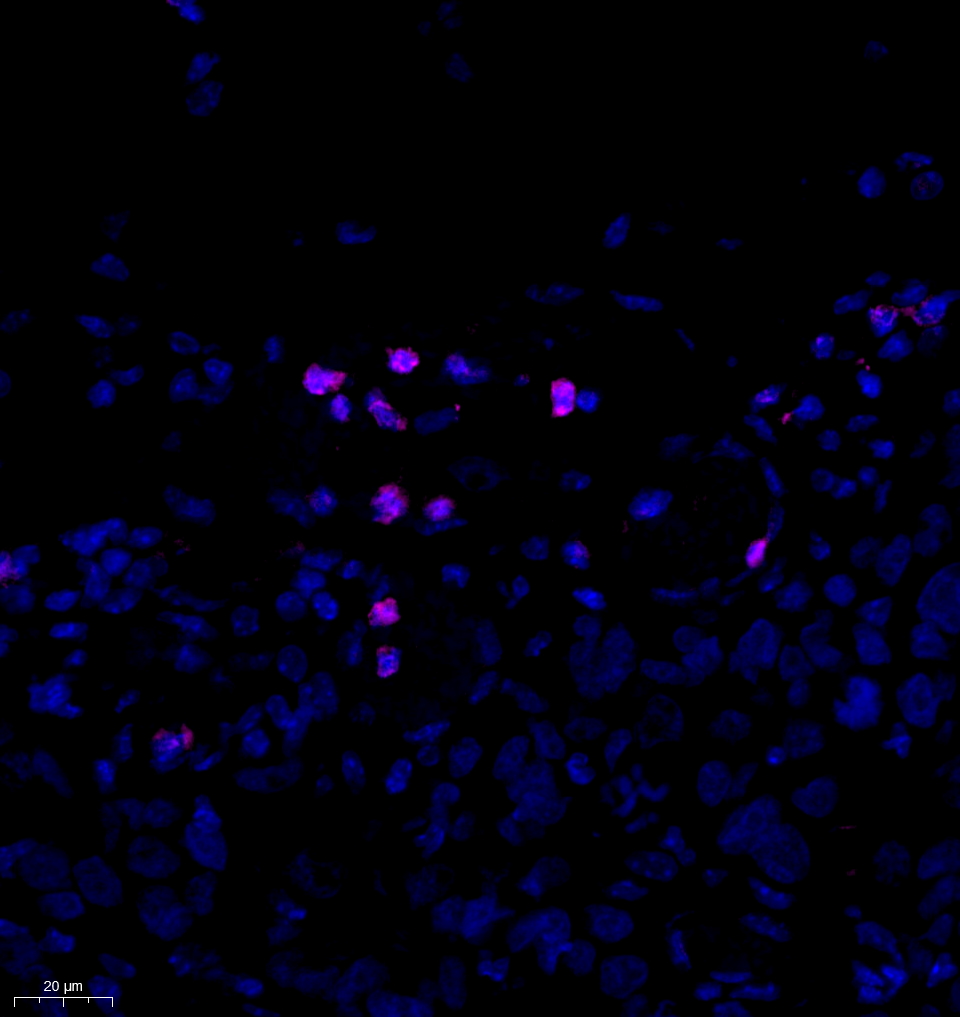


Untreated

Treated

VEGF

| **Slides** | **% IL6**  **Positive Cells** | **%VEGF**  **Positive Cells** |
| --- | --- | --- |
| **Untreated-1** | 0.4 | 3.3 |
| **Untreated-2** | 0.3 | 1.6 |
| **Untreated-3** | 0.5 | 2.0 |
| **AM11095-1** | 1.6 | 2.6 |
| **AM11095-2** | 1.2 | 7.9 |
| **AM11095-3** | 0.8 | 6.9 |

**Figure S5: Immunofluorescence staining of mice tumor sections from untreated mice or AM11095 treated mice.** The sections were incubated with the primary antibody Anti-IL6 (GB11117, 1:100) and anti-VEGF (R&D systems AF293, 1-100) overnight at 4°C. Sections were rinsed with PBS and incubated with Donkey anti-Goat AF488 (Invitrogen, A32814, 1:500) for 1 hour at room temperature followed by 3 times of wash. Subsequently, the sections were with incubate with Goat anti-rabbit AF647 (Invitrogen, A21245, 1:500) and Goat anti-mouse AF555(Invitrogen, A21424, 1:500) for another 1 hour. Lastly, quench autofluorescence with Sudan Black and stain DAPI. Whole slide scanning (40x) was performed on a Panoramic midi scanner (3D histech).
